# Prevalence, underlying causes, and determinants of maternal near miss in Ethiopia: a systematic review and meta-analysis

**DOI:** 10.3389/fmed.2024.1393118

**Published:** 2024-10-08

**Authors:** Neamin Tesfay, Girmay Hailu, Dumesa Begna, Medhanye Habtetsion, Fitsum Taye, Fitsum Woldeyohannes, Ruxana Jina

**Affiliations:** ^1^Centre of Public Health Emergency Management, Ethiopian Public Health Institute, Addis Ababa, Ethiopia; ^2^Felge Meles Primary Hospital, Addis Ababa Health Bureau, Addis Ababa, Ethiopia; ^3^Health Financing Department, Clinton Health Access Initiative, Addis Ababa, Ethiopia; ^4^Data Impact Program, Vital Strategies, New York, NY, United States

**Keywords:** uterine hemorrhage, pregnancy induced hypertension, maternal near miss (MNM), puerperal infection, maternal death surveillance and response

## Abstract

**Background:**

Maternal near miss (MNM) is one of the newly adopted assessment parameters to gauge the quality of maternity care. In Ethiopia, several studies have been conducted to investigate the incidence, underlying causes, and determinants of MNM. However, the findings from those studies vary greatly and are largely inconsistent. Thus, this review aims to more robustly estimate the pooled prevalence, identify underlying causes, and single out determinants of MNM in Ethiopia.

**Methods:**

Studies were searched from international databases (PubMed/ Medline, Cochrane Library, and Embase databases) and other potential sites. All observational studies were included. Heterogeneity between studies was checked using Cochrane Q test statistics and I^2^ test statistics and small study effects were checked using Egger’s statistical test at a 5% significance level. Outcome measures were overall and specific underlying causes (obstetrics hemorrhage, hypertensive disorder pregnancy, pregnancy-related infection) rates of MNMs per 10,000 live births.

**Result:**

The meta-analysis included 43 studies consisting of 77240 MNM cases. The pooled prevalence MNM per 1000 live births in Ethiopia was 54.33 (95% CI: 33.93 to 85.89). Between-study heterogeneity was high (I^2^ = 100%, *P* < 0.0001), with the highest rate observed in Amhara region (384.54 per 1000). The prevalence of obstetrics hemorrhage (14.56 per 1000) was higher than that of hypertensive disorder pregnancy (12.67 per 1000) and pregnancy-related infections (3.55 per 1000) were identified as underlying causes. Various factors, including socio demographic characteristics, previous medical and obstetrics history as well as access to and quality of care obtained, were associated with MNM.

**Conclusion:**

Almost six women encounter near miss among a hundred live births in Ethiopia. Obstetric hemorrhage and hypertensive disorder pregnancy were the most common underlying causes of MNM. Both individual and facility level determinants were found to be associated with MNM. Considering the magnitude and identified factors, tailored measures should be taken at every stage of the continuum of care.

**Systematic review registration:**

https://www.crd.york.ac.uk/prospero/, identifier CRD42023395259.

## Introduction

Maternal near miss (MNM) is a condition where a woman nearly died and survived complications that arise during pregnancy, childbirth, or within 42 days after the termination of pregnancy (1). It is a newly adopted parameter used to evaluate the quality of maternity service and provide additional insight into the overall level of the health system (2, 3).

Globally, similar to maternal deaths, the burden of maternal near miss (MNM) is more prominent in sub-Saharan African countries (4). To obtain comparable estimates, the World Health Organization (WHO) has developed identification criteria to capture MNM cases; however, these criteria have been criticized for underestimating the true burden of MNM in low-resource settings (5–7). Therefore, adapting and modifying the identification tool to the local context would help assess the continuum of care adequately and obtain comprehensive information to gain a clear view of the path to survival (8, 9). Additionally, conducting a review of MNM cases has far-reaching implications because it provides a live example of the quality of care rather than reviewing cases of women who died due to pregnancy-related complications (10). Moreover, routine review of MNM cases not only positively impacts efforts to reduce the burden of maternal mortality but also plays a role in improving prenatal outcomes and reducing treatment costs (11, 12).

Ethiopia is one of the countries with a very high burden of maternal mortality, with a rate of 401 per 100,000 live births in 2017, despite a decline in the last two decades (13, 14). Significant interventions, such as enhancing the health-seeking behaviors of the community, providing trained manpower, and expanding and equipping health facilities, have been implemented to improve maternal and child health outcomes in Ethiopia (15, 16). However, despite these measures, inadequate quality of care, lack of quantified evidence, and insufficient monitoring of the implemented plans have been major obstacles hindering the achievement of targets set to reduce maternal deaths at both the local and global levels (17–20).

Recognizing the gap in evidence generation, following the recommendation of the WHO, Ethiopia has established maternal and perinatal death surveillance and response (MPDSR) systems to prevent future deaths through a continuous action and surveillance cycle (21, 22). However, the system has not been fully implemented due to low community engagement, underreporting, and limited involvement of health professionals, who may avoid participation due to fear, blame, and accountability issues (23, 24). Additionally, the focus of MPDSR solely on mortality, without including severe morbidity, has made it challenging to estimate MNM using routine data (25).

As an alternative approach, the National Emergency Obstetric and Newborn Care (EmONC) assessment was used to measure the burden of MNM in Ethiopia. However, conducting this survey every five years has made it difficult to adapt to annual planning (26). Despite these challenges, the prevalence of MNM in Ethiopia ranges from 13% to 31%, which is higher than that in other sub-Saharan African countries (27, 28).

Similar to maternal death, MNM shares similar pathological and circumstantial factors (1). MNM is considered one of the inequalities within the multidimensional chain of causes that spans from macrosocial factors (such as quality of care and structural and societal factors) to micro clinical factors (including pathways from specific clinical precursors) and specific individual-level factors (29). The major underlying causes of MNM include hypertensive disorders of pregnancy (HDP), obstetric hemorrhage, uterine rupture, abortion-related complications, and malaria, all of which are associated with micro clinical factors (30–32). Individual-level factors related to MNM include maternal age, history of antenatal care (ANC), residence, income, educational status, unemployment, lack of awareness of pregnancy danger signs, and previous medical history [preexisting chronic illness and previous cesarean section (C/S)] (33–36). Macrosocial factors, such as delays in transportation and access to blood products, multiple referrals, and timely treatment after admission, are also significant contributors to MNM (37–40).

Several studies have been conducted on MNM in Ethiopia. Variations were observed in both prevalence and underlying causes across the studies, highlighting inconsistencies and uncertainties, primarily stemming from the failure to consider the most appropriate denominator, which is conspicuously lacking in most reviewed articles (41). This can lead to incorrect estimations and misinterpretations of the findings. Taking all of this into consideration, this review aimed to offer a comprehensive overview of the prevalence, underlying causes, and determinants of MNM in Ethiopia by carefully selecting the most suitable denominator and establishing a temporal trend of it.

## Materials and methods

### Protocol and registration

As depicted in Supplementary Material 1, the updated Preferred Reporting Items for Systematic Reviews and Meta-Analysis (PRISMA) statement, along with and Meta-analysis of Observational Studies in Epidemiology (MOOSE), was used to report this review (42). The protocol has been registered on an International Prospective Register of Systematic Review (PROSPERO), University of York Center for Reviews and Dissemination^1^ (ref no ID: CRD42023395259).

### Study design and search strategy

This review was designed to estimate the incidence, underlying causes, and determinants of MNM among women in Ethiopia. The PubMed/Medline, Cochrane Library, and Embase databases were systematically searched for relevant studies published online from January 2010 to December 2023. The gray literature was retrieved using Google Scholar searches with the inclusion of university repositories. These core search terms and phrases were considered interchangeably in different databases. The details are presented in Supplementary Material 2.

### Eligible criteria

The PECO (population, exposure, comparison, and outcomes) technique, which primarily uses condition, context, and population (CoCoPop) questions, was employed by the authors to determine the inclusion and exclusion criteria for this systematic review and meta-analysis (43, 44) and the detail is annexed in Supplementary Material 3. Studies were included if they (1) were conducted in Ethiopia from January 2010 to December 2023; (2) were observational studies (cross-sectional, cohort, and case controls), (3) published in the English language, (4) defined the criteria of diagnosis of MNM either using WHO or the modified version of sub-Saharan African (SSA) as indicated in Supplementary Material 4 and (5) Both published and unpublished articles and reports were included. Anonymous reports, editorials, case reports, and articles without full access (after contacting the corresponding author two times through e-mail) were excluded from the review.

### Outcome measurement

The primary goal of this review was to quantify the prevalence of MNM, calculated by dividing the number of MNM cases by the total number of live births, and then multiplying by 1000. The secondary objective was to determine the underlying medical causes leading to MNM, also computed by dividing the number of MNM cases with specific underlying causes by the total number of live births, and multiplying by 1000. The third outcome sought to analyze the determinants of MNM, encompassing factors ranging from individual-level to health facility or community-level factors.

### Validity assessment

The quality of each study was assessed by the Joanna Briggs Institute (JBI) quality appraisal checklist designed for various study methodologies (45). These critical appraisal checklists were adapted for the prevalence, cross-sectional, case-control, and cohort studies. After selecting the appropriate checklists, two authors independently reviewed and evaluated the quality of each study using these tools. Disagreements between authors that arose while reviewing the quality were solved based on evidence-based discussions. With all quality assessment items of each study design, studies scores of 5 or more JBI critical appraise were considered as suitable for further analysis (46).

### Data extraction and study selection

After retrieving all studies from the databases, they were imported into the reference manager, EndNote version 16 software, to remove duplicated studies. Afterwards, all eligible studies were examined by the authors based on the title and abstract for possible inclusion. The entire full-text articles were then reviewed to decide on articles to be included. All needed qualitative and quantitative data from the selected studies were extracted independently by the two authors using a predetermined standardized data extraction format. The data abstraction format included primary author, number of liver birth, year of publication, region of study, sample size, study design, study setting, study outcome, diagnosis criteria, and indicators to calculate the prevalence of MNM (MNM cases and the total number of live birth).

### Statistical analysis

Data were extracted in Excel and exported to R software version 4.4.1 (R Project for Statistical Computing). for further analysis. The metaprop function from the R package meta was used to calculate the pooled effect estimates (47). Meta-analysis was performed with the DerSimonian-Laird random-effects model with Hartung-Knapp-Sidik-Jonkman variance correction (48). Individual and pooled estimates were graphically displayed using forest plots. A random-effects model was used to accommodate variation that stemming from study design, sample size, place of study and diagnosis criteria. Between studies heterogeneity was assessed using I^2^ test statistic values, expressed as % (low (25%), moderate (50%), and high (75%) and Cochrane’s Q statistic (significance level < 0.05) (49). To further identify the source of heterogeneity, multivariate meta-regression analysis was conducted. Moreover, a leave-one-out sensitivity analysis was done to point out the influence of a single study on the overall estimate. Finally, Egger’s test was used to assess publication bias objectively and publication bias was declared at a p-value of less than 0.05 (50).

## Result

### Study selection

**FIGURE 1:**
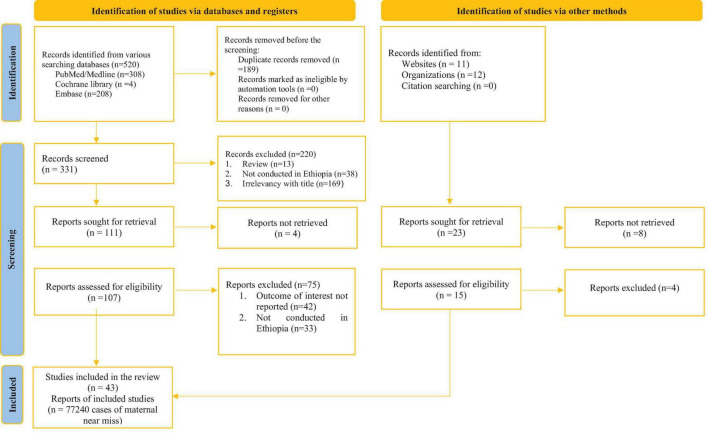
Flow diagram showing the procedures of selecting studies for meta-analysis of the prevalence incidence underlying causes, and determinants of maternal near miss in Ethiopia, 2022 Characteristics of included studies.

A total of 542 articles were retrieved through electronic database searching, out of which 122 underwent full-text screening. Of the included studies, 43 articles met the inclusion criteria to be included in the final review (Figure 1).

Twenty-four cross-sectional, 14 case-control, and 5 cohort studies were included in the review. Regarding the geographical coverage of the review, two studies were conducted at a national level (defined as studies that included more than two regions of the country) (51, 52) and the remaining studies were conducted exclusively in ten regions and city administrations [Addis Ababa = 3 = [(53, 54)], Amhara = 8 (55–62), Central Ethiopia = 5 (63–67), Gambella = 1 (68), Harari = 4 (69–72), Oromia = 12 (73–84), Sidama = 2 (85, 86), South Ethiopia = 2 (87, 88), Southwest Ethiopia Peoples’ Region (SWEPR) = 1 (89), Tigray = 4 (90–93)]. A total of 77,240 cases of MNM were identified with a minimum of 31 cases from Tigray (91) and a maximum of 67,576 cases from a national study (52). Only five studies utilized SSA criteria for the identification of MNM cases while the remaining studies used the WHO criteria. The detail is annexed in Supplementary Material 5.

### Pooled prevalence of maternal near miss

**FIGURE 2:**
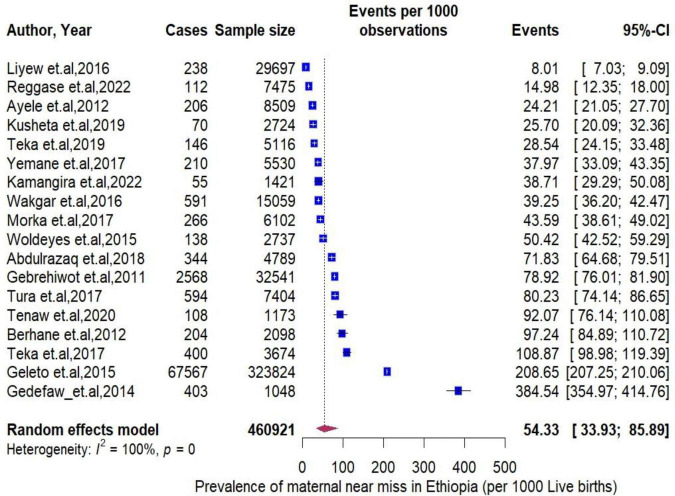
Forest plot showing the pooled prevalence of maternal near miss in Ethiopia.

**FIGURE 3:**
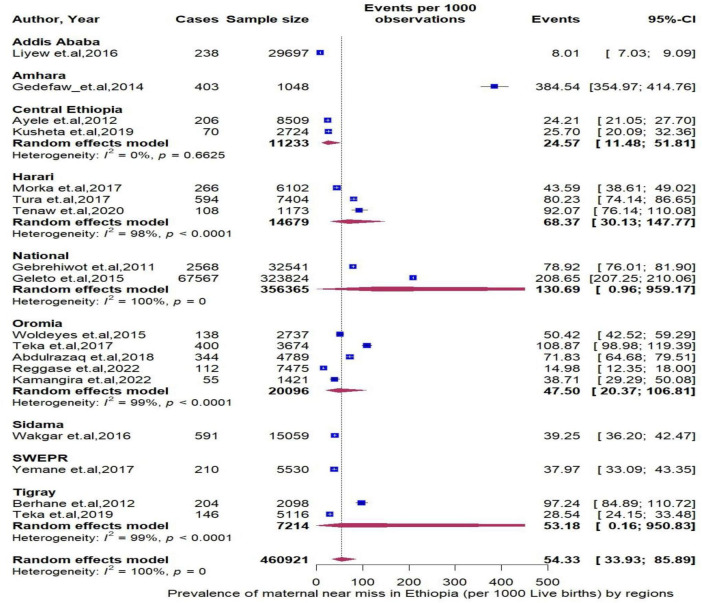
Forest plot showing the pooled prevalence of maternal near miss in Ethiopia stratified by region.

The pooled period prevalence of maternal near miss per 1000 live births was 54.33 (95% CI: 33.93 to 85.89). Between-study heterogeneity was observed (I^2^ = 100%, *P* < 0.0001, Figure 2). To assess regional differences in the pooled rates of MNMs, a subgroup meta-analysis was conducted stratified by region (Figure 3). The region of Amhara demonstrated the highest prevalence of MNMs at 384.54 (95% CI: 354.97 to 354.97, I2 = NA), while the lowest rate was observed in Addis Ababa at 8.01 (95% CI: 7.03 to 9.09, I^2^ = NA).

**FIGURE 4:**
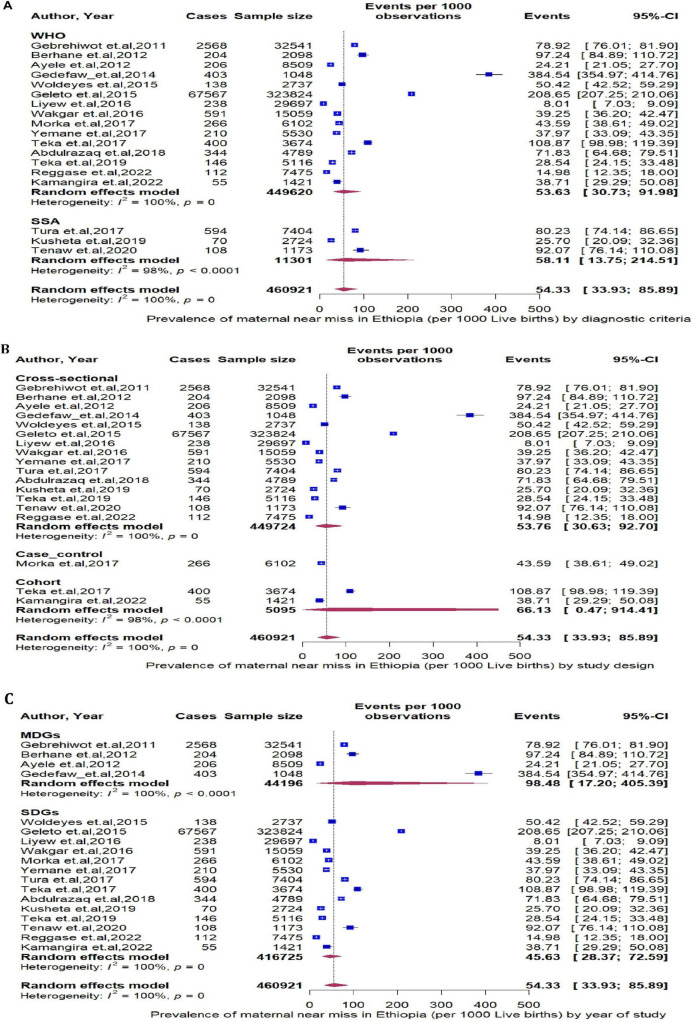
**(A)** The pooled prevalence of maternal near miss stratified by diagnostic criteria. **(B)** The pooled prevalence of maternal near miss stratified by study design. **(C)** The pooled prevalence of maternal near miss stratified by diagnostic year of study.

Additionally, MNM cases diagnosed through SSA criteria had a prevalence of 58.11 (95% CI: 13.75 to 214.51, I^2^ = 98%, Figure 4A), those captured by a cohort study design had a prevalence of 66.13 (95% CI: 0.47 to 914.41, I^2^ = 98%, *P* < 0.0001, Figure 4B), and those identified during the Millennium Development Goals (MDGs) era had a prevalence of 98.48 (95% CI: 17.20 to 405.39, I^2^ = 100%, *P* < 0.0001, Figure 4C), which indicate a higher prevalence of MNMs in these categories compared to their counterpart groups.

### Underlying causes of maternal near miss

**FIGURE 5:**
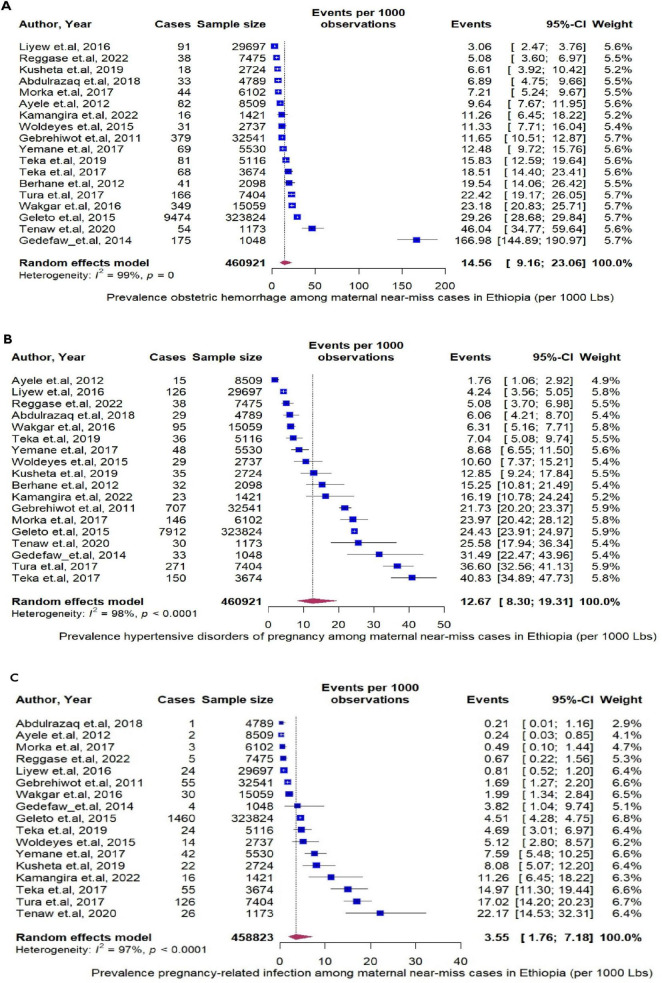
**(A)** The pooled prevalence of obstetrics hemorrhage per 1000 live births. **(B)** The pooled prevalence of by hypertensive disorder of pregnancy per 1000 live births. **(C)** The pooled prevalence of pregnancy related infection per 1000 live births.

**FIGURE 6:**
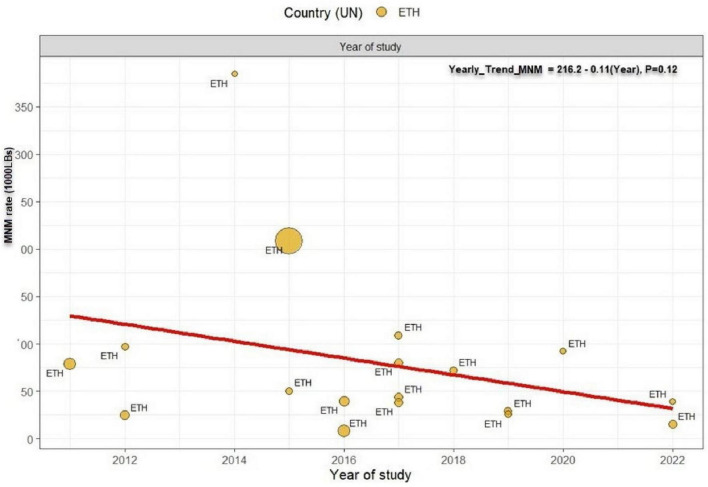
Temporal trend in the prevalence of maternal near miss in Ethiopia. The prevalence of MNM decreased at a rate of 0.9 per year from 2011 to 2022 with *p*-value 0.12. Linear fit from linear regression model. The size of the circle is proportional to the sample size of each study.

Next, we assess specific MNMs underline causes —obstetrics hemorrhage, hypertensive disorder of pregnancy, and pregnancy related infection. Among the MNMs, obstetrics hemorrhage rate was the highest, 14.56 per 1000 (95% CI: 9.16 to 23.06, I^2^ = 99%, *P* < 0.0001, Figure 5A) followed by hypertensive disorder of pregnancy; 12.96 (95% CI: 8.30 to 19.31, I^2^ = 98%, *P* < 0.0001, Figure 5B) and pregnancy related infection were reported less frequently, 3.55 per 1000 (95% CI: 1.76 to 7.18, I^2^ = 97%, *P* < 0.0001, Figure 5C). Using year of study, and starting from 2011, the rate of MNMs deceased by 0.9 % per year (*P* = 0.12) (Figure 6).

### Sensitivity analysis and publication bias

To assess the potential for outlier and influential studies affecting the robustness of the pooled estimates, influence sensitivity analyses was conducted for the prevalence of MNMs (Supplementary Material 6), obstetrics hemorrhage (Supplementary Material 7), hypertensive disorder of pregnancy (Supplementary Material 8) and pregnancy related infection (Supplementary Material 9) separately (94). Trim and fill analysis was conducted to adjust for the potential publication bias. Analyses suggest that the adjusted effect estimates would fall in the range of 83 to 298 per 1000 live births, and 8 additional studies were added to the funnel plots (Supplementary Materials 10, 11). To further identify source of heterogeneity, multivariate meta-regression analysis was conducted. The only significant factor that remained was the study area. Geographically, when compared with a study from Addis Ababa, a high prevalence of maternal near miss was observed from studies conducted in Amhara region [β = 4.26, 95% CI (1.46–7.12)] and at national level [β = 2.88, 95% CI (0.70–5.06)] (Supplementary Material 12).

### Determinants of maternal near miss

Various individual factors (i.e. socio demographic charactertics, previous medical and obstetrics history) were identified as determinants of MNM. These factors include lack of ANC follow-up [AOR = 2.76, 95% CI (1.75–5.06), I^2^ = 92.6%, *P* < 0.0001], rural residency [AOR = 1.96, 95% CI (1.39–2.78), I^2^ = 92.4%, *P* < 0.0001], lack of education [AOR = 2.39, 95% CI (1.69–3.39), I^2^ = 82.3%, *P* < 0.0001], being grand multipara women [AOR = 2.41, 95% CI (1.82–3.20), I^2^ = 66.9%, *P* < 0.0001], having a monthly income ≤ 60 USD [AOR = 1.81, 95% CI (1.07–3.04), I^2^ = 89.4%, *P* = 0.026], preexisting medical conditions [AOR = 6.17, 95% CI (3.77–10.11), I^2^ = 85.3%, *P* < 0.0001], advanced maternal age (above 35) [AOR = 1.90, 95% CI (1.44–2.52), I^2^ = 79.4%, *P* < 0.0001], previous history of C-section [AOR = 3.68, 95% CI (1.95–6.95), I^2^ = 84.8%, *P* < 0.0001], history of female genital mutilation (FGM) [AOR = 1.40, 95% CI (1.02–1.96), I^2^ = 78.5%, *P* = 0.032], and decide to seek care [AOR = 1.80, 95% CI (1.03–3.13), I^2^ = 90.9%, *P* = 0.039].

**FIGURE 7:**
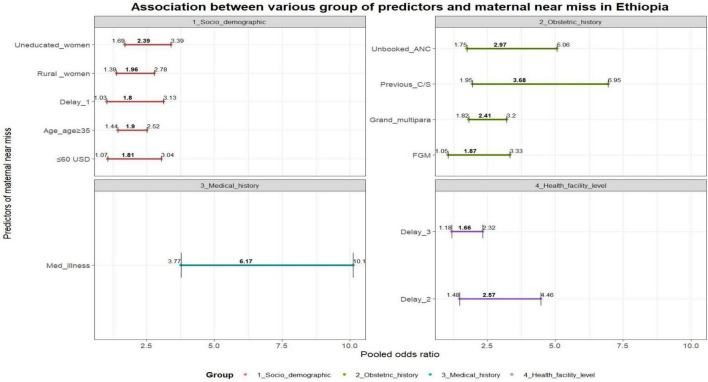
– Association between various group of predictors and maternal near miss in Ethiopia: Delay 1 (delay to decide to seek care), previous C/S (previous cesarian section), FGM (having female genital mutilation), med.illesss (previous chronic medical illness), Delay 2 (delay to reach care), delay 3 (delay to receive care).

On the other hand, a delay in arrival at a health facility [AOR = 2.57, 95% CI (1.48–4.66), I^2^ = 94.8%, *P* < 0.0001] and obtaining inadequate care [AOR = 1.66, 95% CI (1.18–2.32), I^2^ = 48.4%, *P* = 0.003] were facility-level factors that increased the risk of developing MNM (Figure 7).

## Discussion

This review provides deep insight into the incidence of MNM and its underlying causes and determinants. The review also confirmed that the major causes of maternal death had a significant role in MNM. Furthermore, both individual factors (maternal age, maternal parity, educational status, monthly income, preexisting medical illness, delay in deciding to seek care, previous history of C/S, and ANC follow-up) and facility-level factors (delay in arriving at a health facility and delay in receiving care) played a decisive role in having MNM.

The pooled national estimate of MNM in Ethiopia was 54.33 cases per 1000 live births, which was higher than that reported in studies in Egypt (95), Tanzania (96), India (97), and Iraq (98) but lower than that reported in Pakistan (99). Variations in incidence may be due to differences in diagnostic criteria, study settings, and health status. The lack of globally agreed-upon diagnostic criteria complicates cross-country comparisons (100). Laboratory and management-based criteria vary, making comparisons difficult, particularly in resource-constrained countries (101). Significantly, MNM cases have declined during the Sustainable Development Goals (SDG) era compared to the Millennium Development Goals (MDG), reflecting progress made under the national health transformation plan. This underscores the necessity for comprehensive monitoring strategies, including workforce training, health facility upgrades, and heightened community engagement (25). Additionally, the review revealed noticeable regional discrepancies, with lower rates observed in Addis Ababa compared to higher rates in the Amhara region. These variations can be attributed to differences in the quality of care and accessibility, with the capital city often benefiting from superior infrastructure and skilled personnel (26). Therefore, concerted efforts should focus on reducing regional disparities by enhancing the quality of care and improving accessibility to healthcare services. Considering the establishment and integration of MNM surveillance into the existing MPDSR through setting suitable diagnosis criteria in consideration of the local context could facilitate and pave the way to gain a comprehensive picture of maternal severe morbidity from a live witness.

Obstetric hemorrhage, hypertensive disorders of pregnancy (HDP), and pregnancy-related infections are significant causes of MNM. Obstetric hemorrhage has been identified as the leading cause in various countries (102–106). In Ethiopia, factors such as advanced maternal age, lack of antenatal care follow-up, grand multiparity, and prolonged labor increase the risk of postpartum hemorrhage (107, 108). A shortage of blood and blood products in Ethiopia poses challenges in managing cases according to treatment protocols (109, 110), leading to the establishment of mini blood banks at Comprehensive Emergency Obstetric Care (CEmOC) facilities (111). However, the full implementation of these blood banks faces issues related to availability, safety, and quality (112, 113). In summary, targeting high-risk groups and improving access to blood products could help prevent complications from obstetric hemorrhage.

Hypertensive disorders of pregnancy (HDP) have emerged as a significant underlying cause of MNM, second to obstetric hemorrhage. This finding aligns with studies conducted in various countries, including Chad (114), and Brazil (115). In Ethiopia, individual-level factors such as advanced maternal age (> 35), family history of chronic illnesses (hypertension and diabetes mellitus), previous history of HDP, body mass index above 25, and lack of nutritional counseling were identified as risk factors for HDP (116–118). Additionally, health system readiness plays a role in determining outcomes (119), as the lack of essential supplies, medications, weak referral systems, and untrained health professionals hinder the control of HDPs (120). Overall, enhancing prenatal examinations, providing health education, strengthening referral systems, and improving the quality of care are essential measures to mitigate adverse outcomes associated with HDPs.

Pregnancy-related infections are cause of MNM in Ethiopia, which has also been revealed elsewhere (121, 122), with modifiable risk factors, including C/S delivery, premature rupture of membranes, anemia, multiple vaginal examinations, referral from other facilities, and home delivery (123–125). Incomplete monitoring and delayed antibiotic initiation exacerbated outcomes (126). Efforts to increase institutional delivery and health service uptake through local structures and a health extension program have led to a significant rise in institutional deliveries in the past two decades (127). Generally, best practices such as administering prophylactic antibiotics, closely monitoring vital signs, and timely care completion are crucial for managing pregnancy-related infections (128).

The risk of MNM in Ethiopia is increased by unattended ANC follow-up, aligning with studies in Nigeria (129), Sudan (130), and Brazil (131). This is attributed to the limited health information available to unbooked ANC women, hindering access to prenatal examinations for identifying maternal and fetal risk factors (132). Factors influencing ANC uptake in Ethiopia include limited media access, spouses’ education, proximity to health facilities, and region of residence (133–135). Additionally, challenges such as inadequate laboratory tests, long wait times, lack of confidentiality, insufficient counseling, and untrained professionals impede quality care (136–139). Efforts are underway to improve ANC uptake and quality in Ethiopia through initiatives such as behavioral change communication campaigns, creating a woman-friendly environment, and enhancing ANC provider capacity (140, 141). Thus, these efforts should be enhanced to improve the uptake of the service.

Residence, educational status, and monthly income were predictors of MNM in the present study. Women in rural areas with low education and income were more likely to experience MNM than were urban, educated, higher-income women. This aligns with studies in conducted elsewhere (142–146). Education, urban living, and higher income enhance women’s capacity to access and understand health information, leading to better decision-making in maternity care. In response to the socioeconomic context, Ethiopia has implemented a primary healthcare program involving community health workers and established health posts for basic services and education at the grassroot level (147). Therefore, strengthening the primary healthcare system is crucial for increasing access for disadvantaged women.

Older women and grand multiparas have a heightened risk of MNM, as supported by studies from South Africa (148) and Scotland (149). This increased risk is attributed to factors such as postpartum hemorrhage, gestational diabetes, and gestational hypertension due to reduced insulin sensitivity, vascular endothelial dysfunction, and decreased oxytocin receptors (150–152). The risks extend to the fetus, leading to perinatal mortality and preterm birth (153, 154). In Ethiopia, elderly women show low engagement in the continuum of care, with lower acceptance rates of contraceptives, institutional delivery, and ANC follow-up (127). Factors such as low education levels, early marriage, and polygamous marriage contribute to grand multiparity (155). Hence, mothers of advanced age and grand multiparas should be recognized as high-risk groups requiring comprehensive care throughout the continuum of care.

Women with preexisting chronic medical conditions and a history of C/S face a greater risk of MNM than do those without these factors, consistent with the findings of other studies (156–158). Conditions such as diabetes, cardiovascular disease, and previous C/S can complicate pregnancy by impacting hemostasis and increasing the likelihood of gestational diabetes, HDP, uterine rupture, and hemorrhage. In Ethiopia, cephalopelvic disproportion and non-reassuring fetal heart rate patterns are leading indications for C/S (159); however, unnecessary C/S procedures are being performed at private facilities beyond WHO recommendations (160). It is crucial to consider women with these risk factors as a high-risk group and provide specialized care throughout the pregnancy continuum. Governments should also ensure adherence to global C/S guidelines.

This study revealed that a history of FGM increased the risk of MNM, consistent with research in Tanzania and Gambia (161, 162). Long-term complications of FGM, such as scarring, which reduces birth canal elasticity, increase the likelihood of prolonged labor, obstetric issues, and difficult delivery (163, 164). In Ethiopia, FGM remains prevalent in regions such as Somalia and Afar (165, 166), where access to healthcare is limited. This practice is driven by beliefs about reducing sexual activity for marriage prospects (167, 168). Ethiopia has passed laws against FGM due to its health implications (169). In general, special attention during pregnancy can help prevent complications from FGM, and community awareness campaigns are essential.

The study demonstrated that delays in deciding to seek care were positively associated with MNM. This finding aligns with similar results from studies conducted in elsewhere (170). A possible explanation is that women faced severe complications that could have been easily treated early on but were hindered by limited awareness of pregnancy danger signs, decision-making power, and reliance on traditional birth attendants (171, 172). In Ethiopia, factors such as low education levels, residency in pastoralist regions, and lack of nearby health facilities contribute to delays in seeking care (173, 174). In conclusion, the results suggest the need to enhance community awareness of institutional delivery through health extension workers, with a particular focus on engaging male partners to facilitate timely decision-making.

In this study, delays in reaching care and receiving optimal care were found to be positively associated with MNM. Similar findings were reported in studies in similar settings (175, 176). The delay in reaching care is primarily attributed to ineffective referral communication, often compromised by a lack of essential ambulance equipment, infrastructure, governing documents, and trained manpower (177–179). In Ethiopia, the unavailability of skilled health providers, obstetric drugs, and a lack of respectful care at health facilities are the main factors contributing to delay three (180, 181). These findings suggest the need for improvement in the referral system and quality of care to enhance maternal health outcomes.

The main strengths of this review include the consideration of appropriate denominators for estimating prevalence through robust statistical techniques and an extensive literature search from various sources. While the review provides comprehensive insights on maternal near miss (MNM), there are limitations that should be acknowledged before interpreting the results. These limitations include: 1) all studies included were facility-based without any community-based studies, impacting the representativeness of the study; 2) not all regions of the country were covered, affecting the inclusiveness of the study; 3) most included studies were cross-sectional, potentially leading to the influence of confounding variables on the outcome variable; and 4) the presence of heterogeneity in the data obtained from the included studies could be considered another limitation of this study.

## Conclusion

The overall prevalence of MNM in Ethiopia is very high, with obstetric hemorrhage, HDP, and pregnancy-related infections being the leading underlying causes. Sociodemographic, obstetric, and medical history, and facility-level factors all play a role in MNM development. Efforts should focus on improving community health-seeking behavior through ANC follow-up and other communication channels to reduce the burden of MNM. High-risk groups, including older women, grand multipara women, and those with a history of C/S and FGM, require special attention from preconception to postpartum care. Further improvements in the quality of care, including the referral system, provision of optimal care, training professionals, and ensuring the availability of essential drugs and supplies, are essential. Lastly, the establishment and integration of MNM surveillance into the existing MPDSR could have a positive impact on facilitating decision-making by providing robust information.

## Data Availability

The original contributions presented in the study are included in the article/Supplementary material, further inquiries can be directed to the corresponding author.

## Footnotes

1
https://www.crd.york.ac.uk/
