# Supplementary material for: Prevalence, underlying causes, and determinants of maternal near miss in Ethiopia: a systematic review and meta-analysis
Source: Front Med (Lausanne). 2024 Oct 8;11:1393118. doi: 10.3389/fmed.2024.1393118 (PMC11493713; doi:10.3389/fmed.2024.1393118)
Supplement: Supplementary file 1 [file Data_Sheet_1.PDF]

Supplementary documents to

Prevalence, underlying cause and determinants of maternal near miss: A Systematic Review and Meta-Analysis

Neamin Tesfay[1], Girmay Hailu[1], Dumesa Begna[1], Medhanye Habtetsion[1], Fitsum Taye[2], Fitsum Woldeyohannes[3], Ruxana Jina [4]

[1]Centre of Public Health Emergency Management, Ethiopian Public Health Institute, Addis Ababa, Ethiopia

[2]Felge Meles Primary Hospital, Addis Ababa Health Bureau, Addis Ababa, Ethiopia

[3]Health Financing Department, Clinton Health Access Initiative, Addis Ababa, Ethiopia

[4] Data impact program, Vital strategies, New York, USA

Supplementary material S1: 2020 PRISMA Checklist

Section and Topic	Item #	Checklist item	Location where item is reported
**TITLE**			
Title	1	Identify the report as a systematic review.	Page 1
**ABSTRACT**			
Abstract	2	See the PRISMA 2020 for Abstracts checklist.	Page 1&2
**INTRODUCTION**			
Rationale	3	Describe the rationale for the review in the context of existing knowledge.	Page 5
Objectives	4	Provide an explicit statement of the objective(s) or question(s) the review addresses.	Page 5
**METHODS**			
Eligibility criteria	5	Specify the inclusion and exclusion criteria for the review and how studies were grouped for the syntheses.	Page 6
Information sources	6	Specify all databases, registers, websites, organisations, reference lists and other sources searched or consulted to identify studies. Specify the date when each source was last searched or consulted.	Page 5&6
Search strategy	7	Present the full search strategies for all databases, registers and websites, including any filters and limits used.	Page 6
Selection process	8	Specify the methods used to decide whether a study met the inclusion criteria of the review, including how many reviewers screened each record and each report retrieved, whether they worked independently, and if applicable, details of automation tools used in the process.	Page 8
Data collection process	9	Specify the methods used to collect data from reports, including how many reviewers collected data from each report, whether they worked independently, any processes for obtaining or confirming data from study investigators, and if applicable, details of automation tools used in the process.	Page 8
Data items	10a	List and define all outcomes for which data were sought. Specify whether all results that were compatible with each outcome domain in each study were sought (e.g. for all measures, time points, analyses), and if not, the methods used to decide which results to collect.	Page 7&8
	10b	List and define all other variables for which data were sought (e.g. participant and intervention characteristics, funding sources). Describe any assumptions made about any missing or unclear information.	Page 8
Study risk of bias assessment	11	Specify the methods used to assess risk of bias in the included studies, including details of the tool(s) used, how many reviewers assessed each study and whether they worked independently, and if applicable, details of automation tools used in the process.	Page 8
Effect measures	12	Specify for each outcome the effect measure(s) (e.g. risk ratio, mean difference) used in the synthesis or presentation of results.	Page 8&9
Synthesis methods	13a	Describe the processes used to decide which studies were eligible for each synthesis (e.g. tabulating the study intervention characteristics and comparing against the planned groups for each synthesis (item #5)).	Page 8&9
	13b	Describe any methods required to prepare the data for presentation or synthesis, such as handling of missing summary statistics, or data conversions.	Page 8&9
	13c	Describe any methods used to tabulate or visually display results of individual studies and syntheses.	Page 8&9
	13d	Describe any methods used to synthesize results and provide a rationale for the choice(s). If meta-analysis was performed, describe the model(s), method(s) to identify the presence and extent of statistical heterogeneity, and software package(s) used.	Page 8&9
	13e	Describe any methods used to explore possible causes of heterogeneity among study results (e.g. subgroup	Page 8&9

Section and Topic	Item #	Checklist item	Location where item is reported
		analysis, meta-regression).	
	13f	Describe any sensitivity analyses conducted to assess robustness of the synthesized results.	Page 8&9
Reporting bias assessment	14	Describe any methods used to assess risk of bias due to missing results in a synthesis (arising from reporting biases).	Page 8&9
Certainty assessment	15	Describe any methods used to assess certainty (or confidence) in the body of evidence for an outcome.	Page 8&9
**RESULTS**			
Study selection	16a	Describe the results of the search and selection process, from the number of records identified in the search to the number of studies included in the review, ideally using a flow diagram.	Page 10
	16b	Cite studies that might appear to meet the inclusion criteria, but which were excluded, and explain why they were excluded.	Fig 1
Study characteristics	17	Cite each included study and present its characteristics.	S3
Risk of bias in studies	18	Present assessments of risk of bias for each included study.	S3
Results of individual studies	19	For all outcomes, present, for each study: (a) summary statistics for each group (where appropriate) and (b) an effect estimate and its precision (e.g. confidence/credible interval), ideally using structured tables or plots.	Fig 2,4,5,6
Results of syntheses	20a	For each synthesis, briefly summarise the characteristics and risk of bias among contributing studies.	Page 11&13
	20b	Present results of all statistical syntheses conducted. If meta-analysis was done, present for each the summary estimate and its precision (e.g. confidence/credible interval) and measures of statistical heterogeneity. If comparing groups, describe the direction of the effect.	Page 11&13
	20c	Present results of all investigations of possible causes of heterogeneity among study results.	Supp and table 2
	20d	Present results of all sensitivity analyses conducted to assess the robustness of the synthesized results.	Supp
Reporting biases	21	Present assessments of risk of bias due to missing results (arising from reporting biases) for each synthesis assessed.	Supp
Certainty of evidence	22	Present assessments of certainty (or confidence) in the body of evidence for each outcome assessed.	Fig 2,4,5,6
**DISCUSSION**			
Discussion	23a	Provide a general interpretation of the results in the context of other evidence.	Page 21
	23b	Discuss any limitations of the evidence included in the review.	Page 28
	23c	Discuss any limitations of the review processes used.	Page 28
	23d	Discuss implications of the results for practice, policy, and future research.	Page 28
**OTHER INFORMATION**			
Registration and protocol	24a	Provide registration information for the review, including register name and registration number, or state that the review was not registered.	CRD42023395259
	24b	Indicate where the review protocol can be accessed, or state that a protocol was not prepared.	Accessed
	24c	Describe and explain any amendments to information provided at registration or in the protocol.	No done
Support	25	Describe sources of financial or non-financial support for the review, and the role of the funders or sponsors in the review.	NA
Competing interests	26	Declare any competing interests of review authors.	Nill
Availability of data, code and other	27	Report which of the following are publicly available and where they can be found template data collection forms; data extracted from included studies; data used for all analyses; analytic code; any other materials used in the	Provided with reasonable request

Section and Topic	Item #	Checklist item	Location where item is reported
materials		review.	to the author

Supplementary material S2: search strategy used for the systematic and Meta-analysis on prevalence, underlying cause, and determinant of maternal near miss in Ethiopia

((((("epidemiology"[MeSH Subheading] OR "epidemiology"[All Fields] OR "prevalence"[All Fields] OR "prevalence"[MeSH Terms] OR "prevalance"[All Fields] OR "prevalences"[All Fields] OR "prevalence s"[All Fields] OR "prevalent"[All Fields] OR "prevalently"[All Fields] OR "prevalents"[All Fields] OR ("epidemiology"[MeSH Subheading] OR "epidemiology"[All Fields] OR "incidence"[All Fields] OR "incidence"[MeSH Terms] OR "incidences"[All Fields] OR "incident"[All Fields] OR "incidents"[All Fields]) OR ("epidemiologies"[All Fields] OR "epidemiology"[MeSH Subheading] OR "epidemiology"[All Fields] OR "epidemiology"[MeSH Terms] OR "epidemiology s"[All Fields]) OR ("burden"[All Fields] OR "burdened"[All Fields] OR "burdening"[All Fields] OR "burdens"[All Fields])) AND (("associate"[All Fields] OR "associated"[All Fields] OR "associates"[All Fields] OR "associating"[All Fields] OR "association"[MeSH Terms] OR "association"[All Fields] OR "associations"[All Fields]) AND ("factor"[All Fields] OR "factor s"[All Fields] OR "factors"[All Fields]))) OR ("analysis"[MeSH Subheading] OR "analysis"[All Fields] OR "determination"[All Fields] OR "determinant"[All Fields] OR "determinants"[All Fields] OR "determinate"[All Fields] OR "determinated"[All Fields] OR "determinates"[All Fields] OR "determinating"[All Fields] OR "determinations"[All Fields] OR "determine"[All Fields] OR "determined"[All Fields] OR "determines"[All Fields] OR "determining"[All Fields]) OR ("risk factors"[MeSH Terms] OR ("risk"[All Fields] AND "factors"[All Fields]) OR "risk factors"[All Fields])) AND (("maternally"[All Fields] OR "maternities"[All Fields] OR "maternity"[All Fields] OR "mothers"[MeSH Terms] OR "mothers"[All Fields] OR "maternal"[All Fields]) AND "near"[All Fields] AND "miss"[All Fields])) OR (("sever"[All Fields] OR "severe"[All Fields] OR "severed"[All Fields] OR "severely"[All Fields] OR "severer"[All Fields] OR "severes"[All Fields] OR "severing"[All Fields] OR "severities"[All Fields] OR "severity"[All Fields] OR "severs"[All Fields]) AND ("maternally"[All Fields] OR "maternities"[All Fields] OR "maternity"[All Fields] OR "mothers"[MeSH Terms] OR "mothers"[All Fields] OR "maternal"[All Fields]) AND ("outcome"[All Fields] OR "outcomes"[All Fields])) OR (("sever"[All Fields] OR "severe"[All Fields] OR "severed"[All Fields] OR "severely"[All Fields] OR "severer"[All Fields] OR "severes"[All Fields] OR "severing"[All Fields] OR "severities"[All Fields] OR "severity"[All Fields] OR "severs"[All Fields]) AND ("maternally"[All Fields] OR "maternities"[All Fields] OR "maternity"[All Fields] OR "mothers"[MeSH Terms] OR "mothers"[All Fields] OR "maternal"[All Fields]) AND ("epidemiology"[MeSH Subheading] OR "epidemiology"[All Fields] OR "morbidity"[All Fields] OR "morbidity"[MeSH Terms] OR "morbid"[All Fields] OR "morbidities"[All Fields] OR "morbids"[All Fields]))) AND ("ethiopia"[MeSH Terms] OR "ethiopia"[All Fields] OR "ethiopia s"[All Fields])

**Result:**
**308**

Translations:

**Prevalence**	"epidemiology"[Subheading] OR "epidemiology"[All Fields] OR "prevalence"[All Fields] OR "prevalence"[MeSH Terms] OR "prevalance"[All Fields] OR "prevalences"[All Fields] OR "prevalence's"[All Fields] OR "prevalent"[All Fields] OR "prevalently"[All Fields] OR "prevalents"[All Fields]
**incidence**	"epidemiology"[Subheading] OR "epidemiology"[All Fields] OR "incidence"[All Fields] OR "incidence"[MeSH Terms] OR "incidences"[All Fields] OR "incident"[All Fields] OR "incidents"[All Fields]
**epidemiology**	"epidemiologies"[All Fields] OR "epidemiology"[Subheading] OR "epidemiology"[All Fields] OR "epidemiology"[MeSH Terms] OR "epidemiology's"[All Fields]
**burden**	"burden"[All Fields] OR "burdened"[All Fields] OR "burdening"[All Fields] OR "burdens"[All Fields]
**associated**	associate"[All Fields] OR "associated"[All Fields] OR "associates"[All Fields] OR "associating"[All Fields] OR "association"[MeSH Terms] OR "association"[All Fields] OR "associations"[All Fields]
**factors**	"factor"[All Fields] OR "factor's"[All Fields] OR "factors"[All Fields]
**determinants**	"analysis"[Subheading] OR "analysis"[All Fields] OR "determination"[All Fields] OR "determinant"[All Fields] OR "determinants"[All Fields] OR "determinate"[All Fields] OR "determinated"[All Fields] OR "determinates"[All Fields] OR "determinating"[All Fields] OR "determinations"[All Fields] OR "determine"[All Fields] OR "determined"[All Fields] OR "determines"[All Fields] OR "determining"[All Fields]

**risk factors**	"risk factors"[MeSH Terms] OR ("risk"[All Fields] AND "factors"[All Fields]) OR "risk factors"[All Fields]
maternal	"maternally"[All Fields] OR "maternities"[All Fields] OR "maternity"[All Fields] OR "mothers"[MeSH Terms] OR "mothers"[All Fields] OR "maternal"[All Fields]
severe	"sever"[All Fields] OR "severe"[All Fields] OR "severed"[All Fields] OR "severely"[All Fields] OR "severer"[All Fields] OR "severes"[All Fields] OR "severing"[All Fields] OR "severities"[All Fields] OR "severity"[All Fields] OR "severs"[All Fields]
maternal	"maternally"[All Fields] OR "maternities"[All Fields] OR "maternity"[All Fields] OR "mothers"[MeSH Terms] OR "mothers"[All Fields] OR "maternal"[All Fields]
outcome	outcome"[All Fields] OR "outcomes"[All Fields]
severe	"sever"[All Fields] OR "severe"[All Fields] OR "severed"[All Fields] OR "severely"[All Fields] OR "severer"[All Fields] OR "severes"[All Fields] OR "severing"[All Fields] OR "severities"[All Fields] OR "severity"[All Fields] OR "severs"[All Fields]
maternal	"maternally"[All Fields] OR "maternities"[All Fields] OR "maternity"[All Fields] OR "mothers"[MeSH Terms] OR "mothers"[All Fields] OR "maternal"[All Fields]
morbidity	"epidemiology"[Subheading] OR "epidemiology"[All Fields] OR "morbidity"[All Fields] OR "morbidity"[MeSH Terms] OR "morbid"[All Fields] OR "morbidities"[All Fields] OR "morbids"[All Fields]

Ethiopia	"ethiopia"[MeSH Terms] OR "ethiopia"[All Fields] OR "ethiopia's"
**Database:**	
PMC	

Supplementary material S3: Searching strategy used based on a variation of a PICOS statement

Population	Women who were in pregnancy, childbirth, or postpartum period in Ethiopia.
Intention	The exposure spans across from the micro clinical factors up to macro social factors. The underline causes leading to MNM such as HDP, obstetrics hemorrhage, uterine rupture, abortion-related complications, and malaria were included as micro micro-clinical factors. Whereas, maternal age, maternal education, monthly income, previous medical history, history of ANC follow-up, and awareness of danger signs of pregnancy were included under individual-level factors contributing to MNM. While delay in reaching care and delay in receiving optimal care were included under macro social factors.
Comparison	Comparing women who were diagnosis with near miss in related to healthy women
Outcome	Prevalence, underlying causes, and determinants of MNM among women.

Supplementary material S4. World Health Organization (WHO) and Sub-Saharan African MNM criteria

World Health Organization maternal near miss criteria		
**Clinical criteria**	**Laboratory-based criteria**	**Management-based criteria**
⇒ Acute cyanosis<br>⇒ Loss of consciousness lasting > 12 h<br>⇒ Gasping<br>⇒ Loss of consciousness and absence of pulse/heartbeat<br>⇒ Respiratory rate > 40 or < 6/min<br>⇒ Stroke<br>⇒ Shock<br>⇒ Uncontrollable fit/total paralysis<br>⇒ Oliguria nonresponsive to fluids or diuretics<br>⇒ Jaundice in the presence of pre-eclampsia<br>⇒ Clotting failure	⇒ Oxygen saturation < 90% for > 60 min<br>⇒ pH < 7.1<br>⇒ PaO2/FiO2 < 200 mmHg<br>⇒ Lactate > 5<br>⇒ Creatinine > 300 mmol/l or > 3.5 mg/dl<br>⇒ Acute thrombocytopenia (< 50, 000 platelets)<br>⇒ Bilirubin > 100 mmol/l or > 6.0 mg/dl<br>⇒ Loss of consciousness and the presence of glucose and ketoacids in urine	⇒ Use of continuous vasoactive drugs<br>⇒ Intubation and ventilation for > 60 min not related to anesthesia<br>⇒ Hysterectomy following infection or hemorrhage<br>⇒ Dialysis for acute renal failure<br>⇒ Transfusion of 5 units of red cell transfusion<br>⇒ Cardio-pulmonary resuscitation (CPR)
**Sub-Saharan African MNM criteria**		
⇒ Acute cyanosis<br>⇒ Gasping<br>⇒ Respiratory rate > 40 or < 6/min<br>⇒ Shock<br>⇒ Oliguria nonresponsive to fluids or diuretics<br>⇒ Failure to form clots<br>⇒ Loss of consciousness lasting ⩾ 12 hf<br>⇒ Cardiac arrest<br>⇒ Stroke	⇒ Oxygen saturation < 90% for > 60 min<br>⇒ Creatinine ⩾ 300 μmol/L or ⩾ 3.5 mg/dL<br>⇒ Acute thrombocytopenia (<50, 000 platelets/mL)<br>⇒ Loss of consciousness and ketoacids in urine	⇒ Hysterectomy following infection or hemorrhage<br>⇒ Transfusion of ⩾ 2 units of red blood cells<br>⇒ Intubation and ventilation for 60 min not related to anesthesia<br>⇒ Cardiopulmonary resuscitation<br>⇒ Laparotomy other than for cesarean section

⇒ Uncontrollable fit/total paralysis<br>⇒ Jaundice in the presence of pre-eclampsia<br>⇒ Eclampsia<br>⇒ Uterine rupture<br>⇒ Sepsis or severe systemic infection<br>⇒ Pulmonary edema<br>⇒ Severe abortion complications<br>⇒ Severe malaria<br>⇒ Severe pre-eclampsia with ICU admission		

Supplementary material S5: Characteristics of included studies.

Prevalence, underlying causes, and determinant of maternal near miss in Ethiopia; A systematic review and metanalysis

S.No	Author	Year	Region	Study setting	study design	MNM	N	Sample size	Obs.hemmo	HTN	Infection	Criteria inclusion	Primary _study outcome	JBI quality status
1	Erega et.al	2021	Amhara	Facility base	Case control	76		304				SSA	Determinants	6
2	Gedefaw_et.al	2014	Amhara	Facility base	Cross-sectional	403	1048	1355	175	33	4	WHO	Prevalence	7
3	Abdulrazaq	2018	Oromia	Facility base	Case control	344		1272	33	29	1	WHO	Determinants	6
4	Habte et.al	2020	Central Ethiopia	Facility base	Case control	81		322	41	18	19	WHO	Determinants	7
5	Demmen et.al	2019	Gambella	Facility base	Case control	103		308	46	40	10	WHO	Determinants	6
6	Mekango et.al	2016	Tigray	Facility base	Case control	103		308	46	40	10	WHO	Determinants	6
7	Kumela et.al	2018	Oromia	Facility base	Case control	61		183	24	25	3	WHO	Determinants	7
8	Teshome et.al	2020	Amhara	Facility base	Case control	88		264				WHO	Determinants	7
9	Tenaw et.al	2020	Harari	Facility base	Case control	108		432				SSA	Determinants	7
10	Liyew et.al	2016	Addis Ababa	Facility base	Case control	216		864				WHO	Determinants	7
11	Aliyi et.al	2019	Oromia	Facility base	Cohort	236		354	39	78	12	WHO	Determinants	7
12	Kebede et.al	2014	South Ethiopia	Facility base	Cohort	96		1440	28	26	13	WHO	Determinants	6
13	Dessalegn et.al	2019	Oromia	Facility base	Case control	80		320	26	25	3	WHO	Determinants	6
14	Mekonnen et.al	2019	Oromia	Facility base	Cross-sectional	85		296	22	48	15	WHO	Prevalence	7
15	Gebrehiwot et.al	2011	National	Facility base	Cross-sectional	2568	32541	35047	379	707	55	WHO	Prevalence	8
16	Morka et.al	2017	Harari	Facility base	Case control	266	6102	306	44	146	3	WHO	Determinants	6
17	Kusheta et.al	2019	Central Ethiopia	Facility base	Case control	70		279				SSA	Determinants	7
18	Liyew et.al	2016	Addis Ababa	Facility base	Cross-sectional	238	29697	29697	91	126	24	WHO	Prevalence	7
19	Woldeyes et.al	2015	Oromia	Facility base	Cross-sectional	138	2737	364	31	29	14	WHO	Prevalence	7

20	Morka et.al	2022	Oromia	Facility base	Cohort	112		335				WHO	Determinants	7
21	Geleto et.al	2015	National	Facility base	Cross-sectional	67567	323824	78195	9474	7912	1460	WHO	Prevalence	8
22	Yemane et.al	2017	SWEPR	Facility base	Cross-sectional	210	5530	845	69	48	42	WHO	Prevalence	7
23	Weldemariam et.al	2022	Tigray	Facility base	Cross-sectional	31		422	15	11	5	WHO	Determinants	5
24	Reggase et.al	2022	Oromia	Facility base	Cross-sectional	112	7475	327	38	38	5	WHO	Prevalence	6
25	Tenaw et.al	2020	Harari	Facility base	Cross-sectional	108	1173	1214	54	30	26	SSA	Determinants	7
26	Kusheta et.al	2019	Central Ethiopia	Facility base	Cross-sectional	70	2724	279	18	35	22	SSA	Prevalence	5
27	Rozina et.al	2021	Amhara	Facility base	Cross-sectional	211		832	8	36	16	WHO	Determinants	6
28	Kamangira et.al	2022	Oromia	Facility base	Cohort	55	1421	117	16	23	16	WHO	Prevalence	7
29	Wakgar et.al	2016	Sidama	Facility base	Cross-sectional	591	15059	15059	349	95	30	WHO	Prevalence	6
30	Abdulrazak et.al	2018	Oromia	Facility base	Cross-sectional	344		1667				WHO	Prevalence	5
31	Teka et.al	2019	Tigray	Facility base	Cross-sectional	146	5116	691	81	36	24	WHO	Prevalence	7
32	Fenta et.al	2017	Amhara	Facility base	Cross-sectional	116		501	36	32	19	WHO	Prevalence	6
33	Berhane et.al	2012	Tigray	Facility base	Cross-sectional	204	2098	2107	41	32		WHO	Prevalence	5
34	Kasahun et.al	2017	Central Ethiopia	Facility base	Case control	77		229	20	13	7	WHO	Determinants	7
35	Dana et.al	2017	South Ethiopia	Facility base	Cross-sectional	146		508				WHO	Prevalence	5
36	Danasu et.al	2019	Oromia	Facility base	Case control	166		664	52	37	20	WHO	Determinants	7
37	Tolosa et.al	2020	Sidama	Facility base	Cross-sectional	51		316	10	21	12	WHO	Prevalence	6
38	Ayele et.al	2012	Central Ethiopia	Facility base	Cross-sectional	206	8509	8989	82	15	2	WHO	Prevalence	6
39	Dile et.al	2013	Amhara	Facility base	Cross-sectional	186		806	51	44		WHO	Determinants	5
40	Asaye et.al	2019	Amhara	Facility base	Cross-sectional	48		303				WHO	Determinants	5
41	Tura et.al	2017	Harari	Facility base	Cross-sectional	594	7404	1054	166	271	126	SSA	Prevalence	8
42	Teka et.al	2017	Oromia	Facility base	Cohort	400	3674	3835	68	150	55	WHO	Prevalence	6
43	Gebremariam et.al	2017	Amhara	Facility base	Cross-sectional	129		905	34	40	16	WHO	Prevalence	6

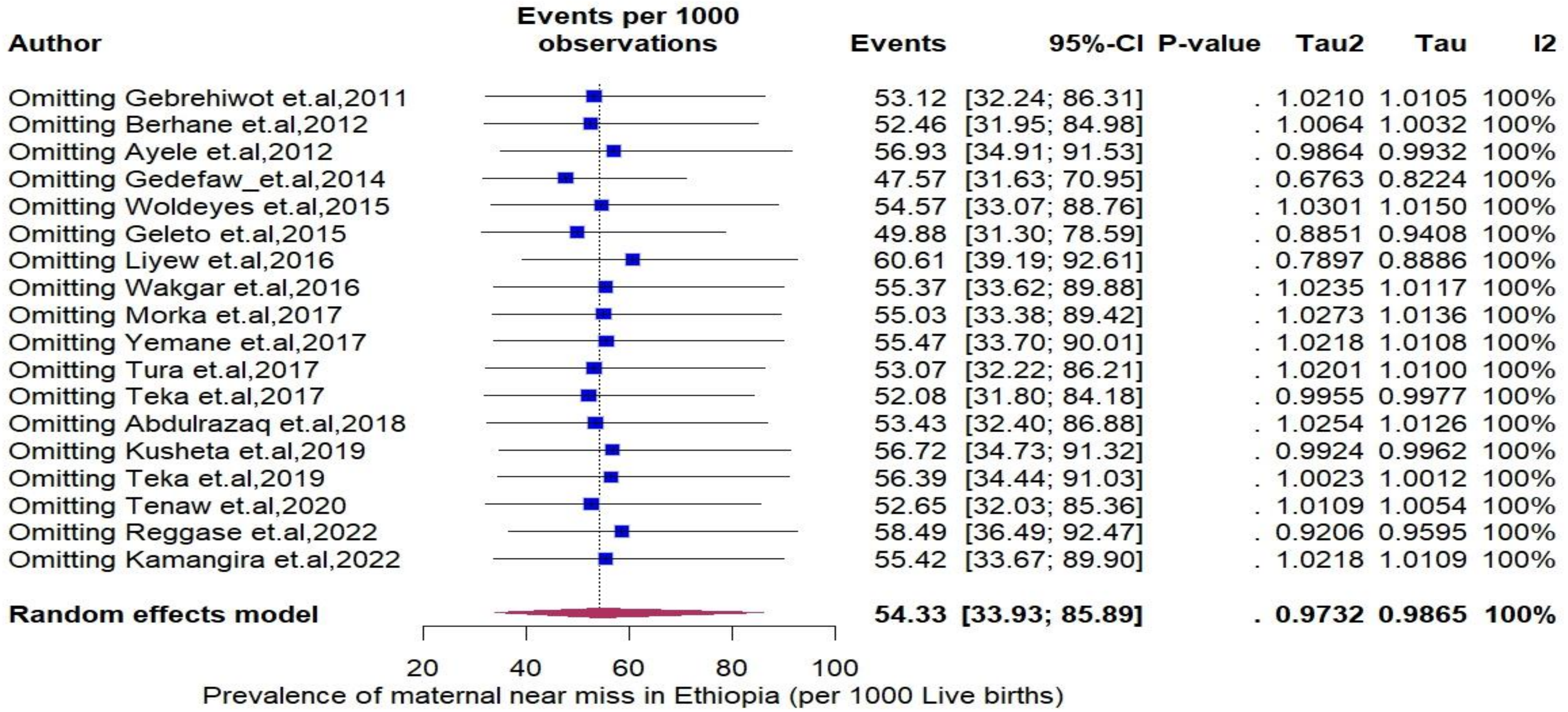

Supplementary material S6: Influence and outlier (leave-one-out meta-analysis) analysis for the prevalence of maternal near miss. The results of our outlier and influence analysis show the recalculated pooled point estimate ranged from 47 to 60 per 1000 livebirth when one study omitted each time.

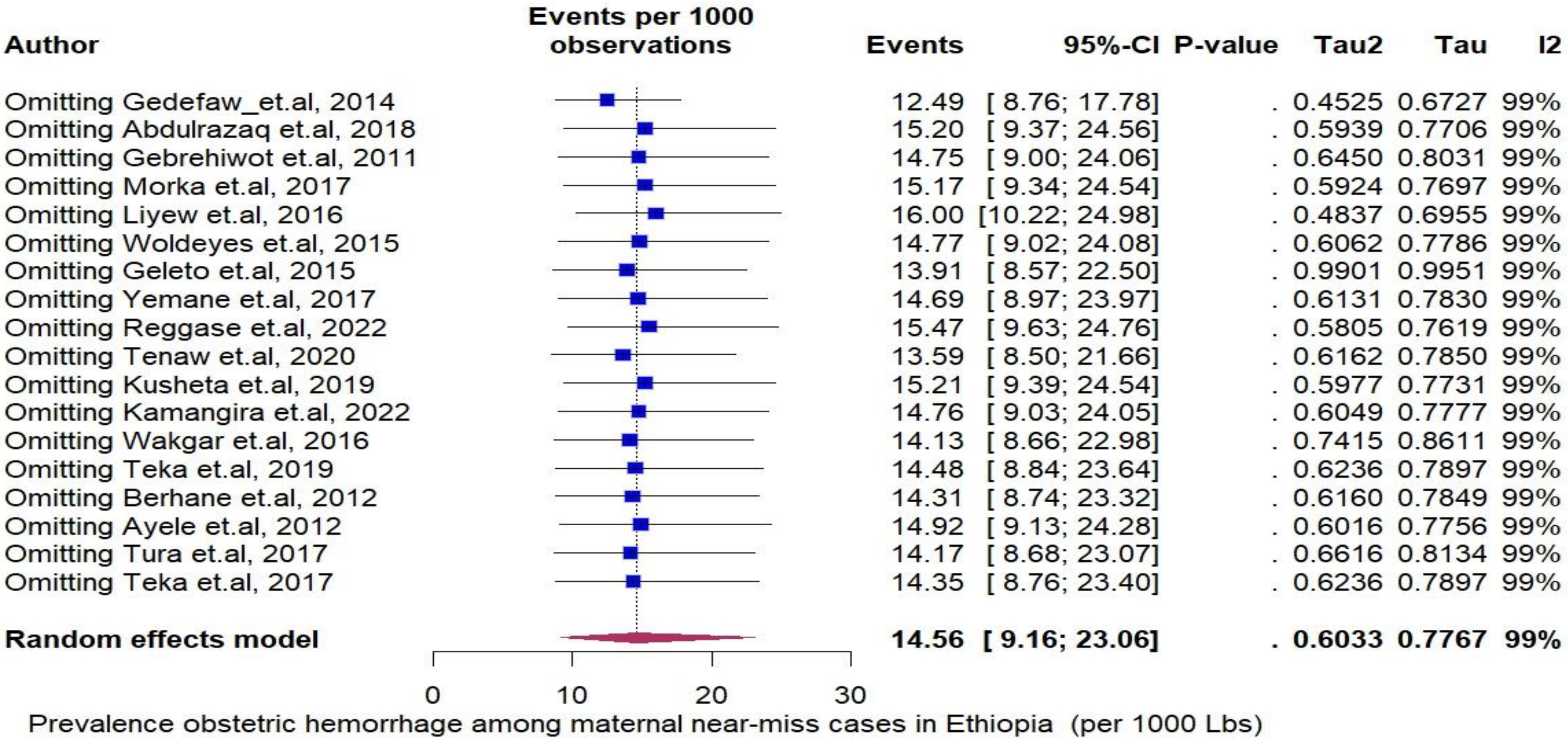

Supplementary material S7: Influence and outlier (leave-one-out meta-analysis) analysis for the prevalence of obstetrics hemorrhage among maternal near miss in Ethiopia. The results of our outlier and influence analysis show the recalculated pooled point estimate ranged from 12 to 16 per 1000 livebirth when one study omitted each time.

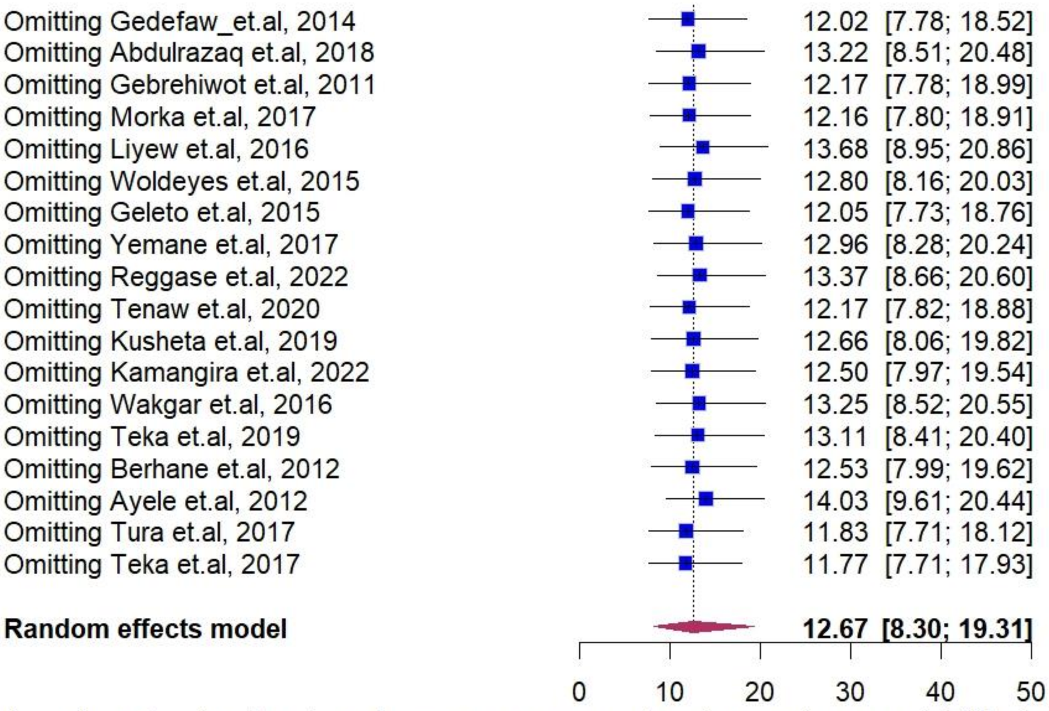

Author	Events per 1000 observations	Events	95%-CI	P-value	Tau2	Tau	I2
Omitting Gedefaw_et.al, 2014		12.02	[7.78; 18.52]	.	0.3285	0.5731	98%
Omitting Abdulrazaq et.al, 2018		13.22	[8.51; 20.48]	.	0.3121	0.5586	98%
Omitting Gebrehiwot et.al, 2011		12.17	[7.78; 18.99]	.	0.5003	0.7073	98%
Omitting Morka et.al, 2017		12.16	[7.80; 18.91]	.	0.3499	0.5915	98%
Omitting Liyew et.al, 2016		13.68	[8.95; 20.86]	.	0.2218	0.4709	98%
Omitting Woldeyes et.al, 2015		12.80	[8.16; 20.03]	.	0.3232	0.5685	98%
Omitting Geleto et.al, 2015		12.05	[7.73; 18.76]	.	0.6180	0.7861	98%
Omitting Yemane et.al, 2017		12.96	[8.28; 20.24]	.	0.3171	0.5631	98%
Omitting Reggase et.al, 2022		13.37	[8.66; 20.60]	.	0.3021	0.5497	98%
Omitting Tenaw et.al, 2020		12.17	[7.82; 18.88]	.	0.3289	0.5735	98%
Omitting Kusheta et.al, 2019		12.66	[8.06; 19.82]	.	0.3260	0.5710	98%
Omitting Kamangira et.al, 2022		12.50	[7.97; 19.54]	.	0.3269	0.5718	98%
Omitting Wakgar et.al, 2016		13.25	[8.52; 20.55]	.	0.2866	0.5353	98%
Omitting Teka et.al, 2019		13.11	[8.41; 20.40]	.	0.3136	0.5600	98%
Omitting Berhane et.al, 2012		12.53	[7.99; 19.62]	.	0.3276	0.5724	98%
Omitting Ayele et.al, 2012		14.03	[9.61; 20.44]	.	0.2948	0.5429	98%
Omitting Tura et.al, 2017		11.83	[7.71; 18.12]	.	0.3528	0.5939	98%
Omitting Teka et.al, 2017		11.77	[7.71; 17.93]	.	0.3331	0.5771	98%
**Random effects model**		**12.67**	**[8.30; 19.31]**	**.**	**0.3237**	**0.5690**	**98%**

Supplementary material S8 : Influence and outlier (leave-one-out meta-analysis) analysis for the prevalence of hypertensive disorder of pregnancy among maternal near miss in Ethiopia. The results of our outlier and influence analysis show the recalculated pooled point estimate ranged from 12 to 14 per 1000 livebirth when one study omitted each time.

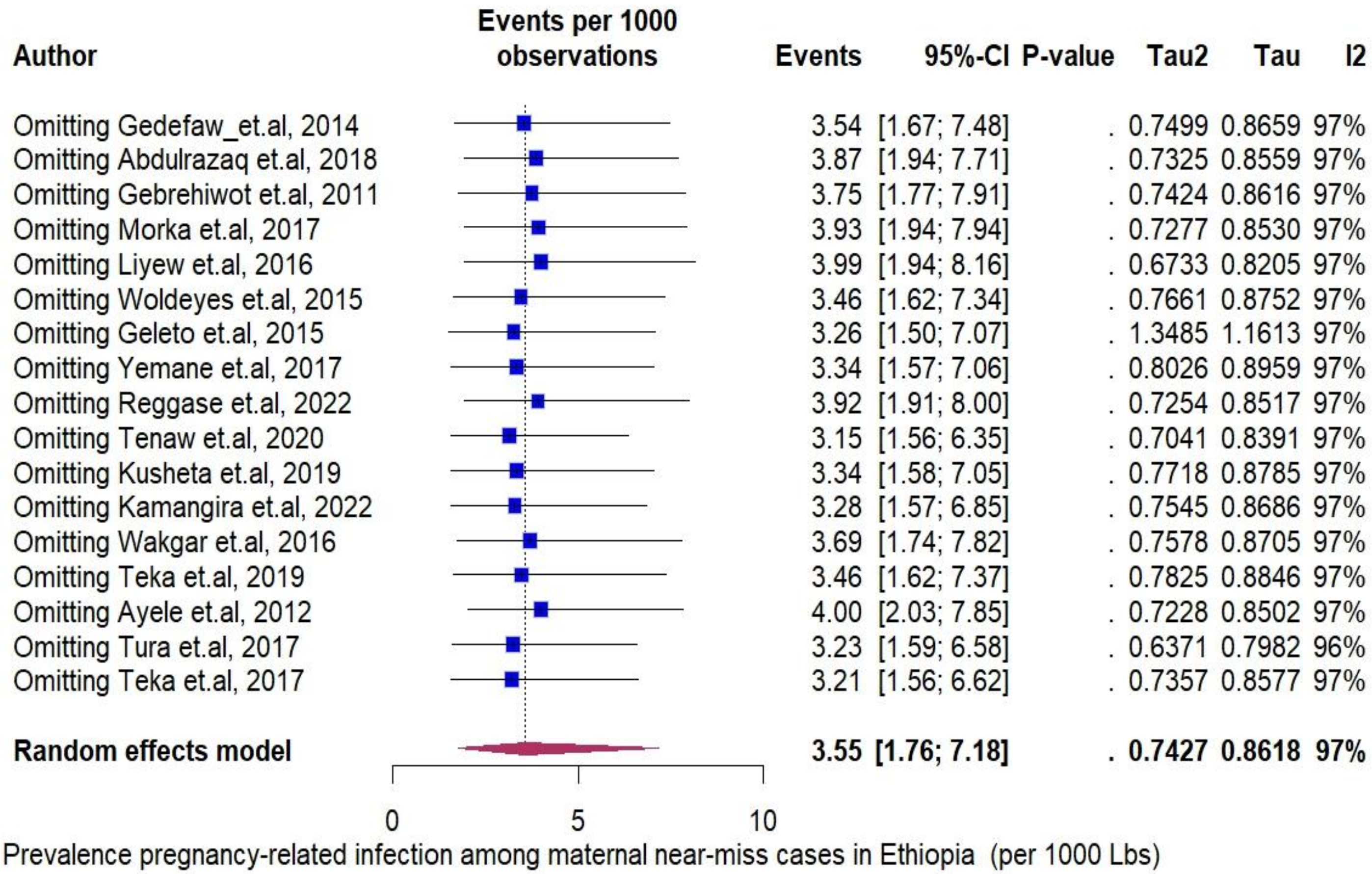

Supplementary material S9: Influence and outlier (leave-one-out meta-analysis) analysis for the prevalence of pregnancy related infection among maternal near miss in Ethiopia. The results of our outlier and influence analysis show the recalculated pooled point estimate ranged from 3 to 4 per 1000 livebirth when one study omitted each time.

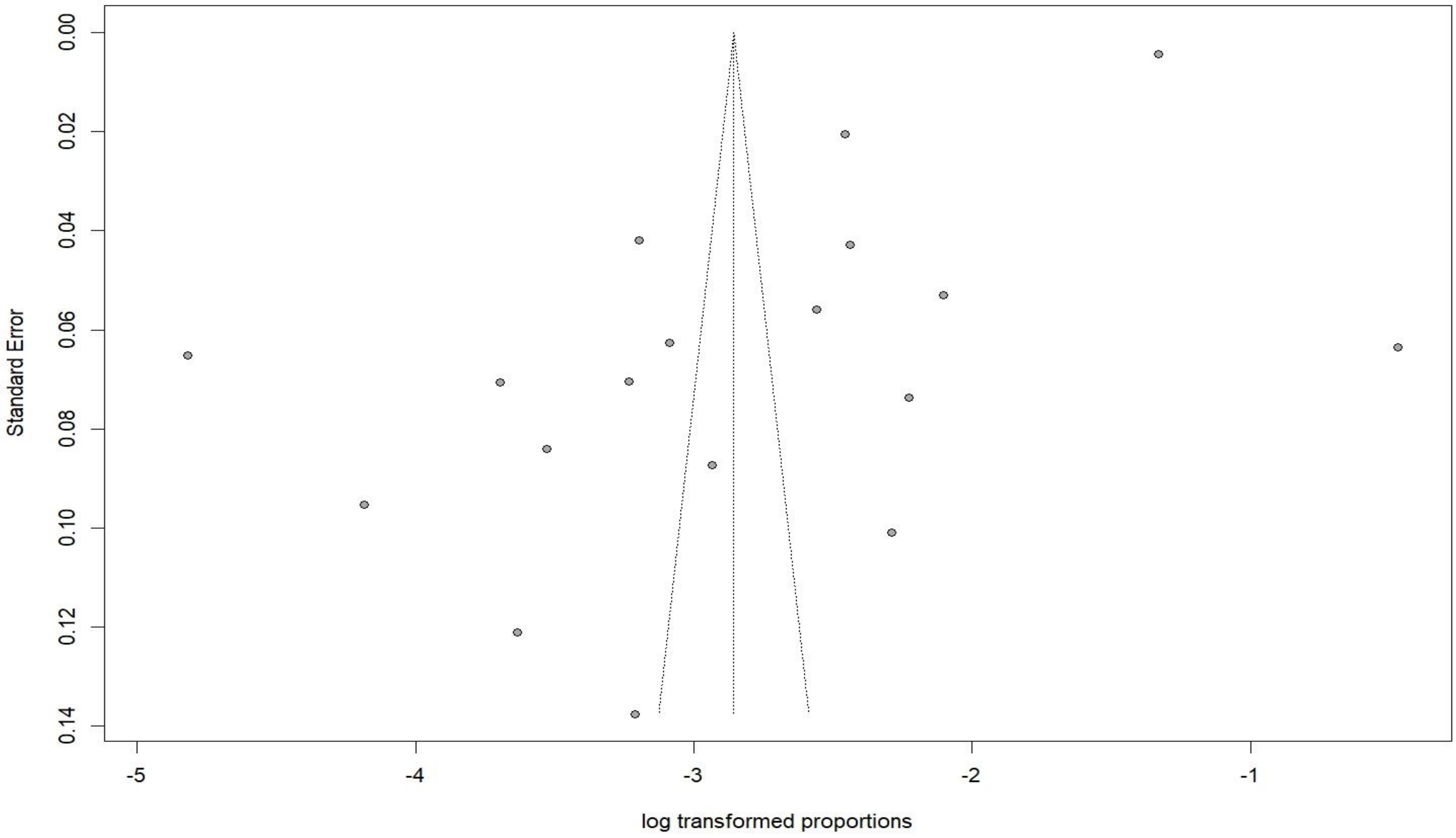

Supplementary material S10: Funnel plots to assess potential for small-study publication bias. Asymmetrical inverted funnel plot suggested presence of publication bias.

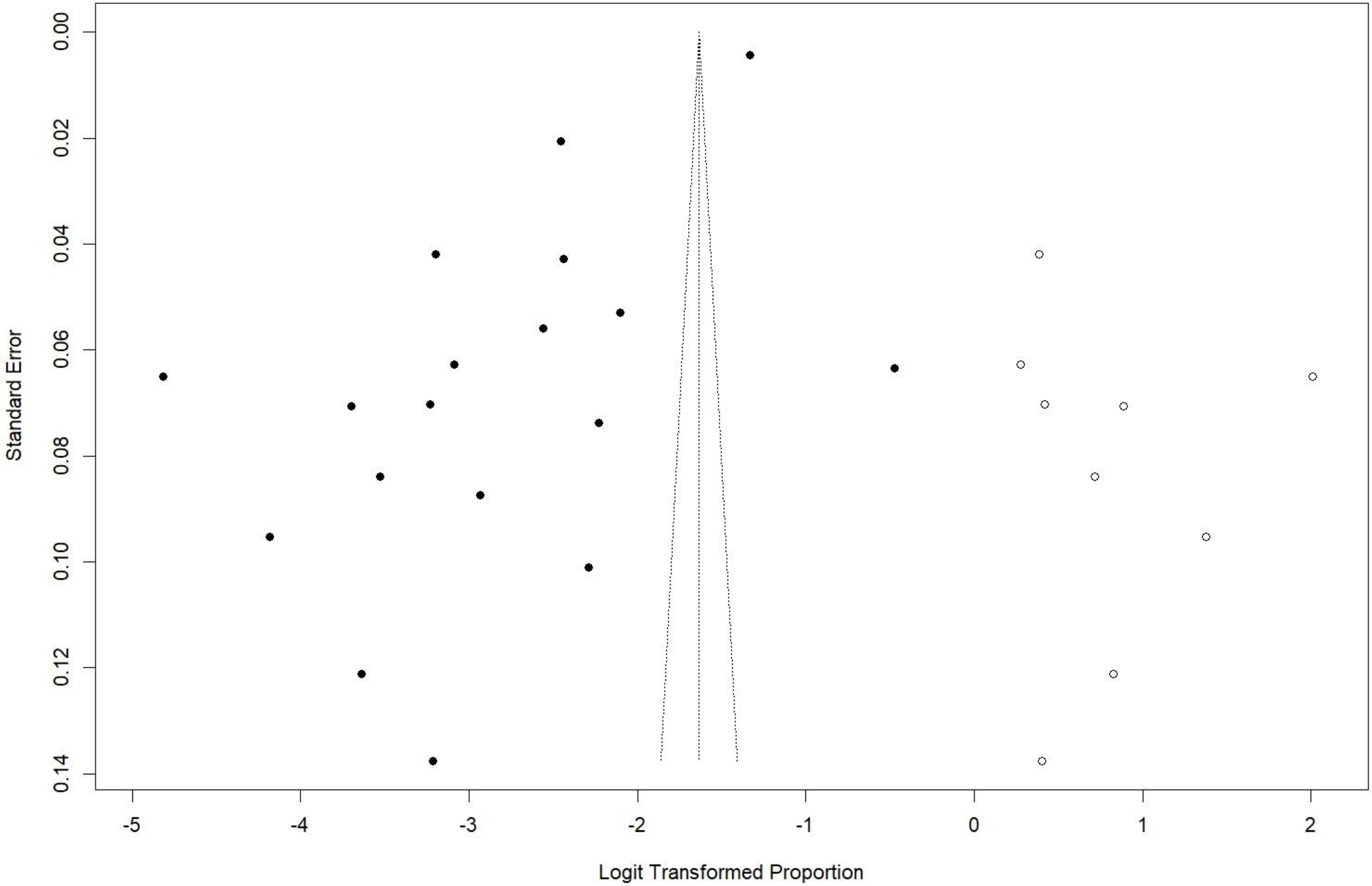

Supplementary material S11: Funnel plots from trim and fill analysis. Duval & Tweedie trim and fill analytical method suggests that the adjusted effect estimates would fall in the range of 83 to 298 per 1000 births and 8 additional studies were added (open circles).

Supplementary material S12: Meta-regression analysis of factors affecting between-study heterogeneity

Source of Heterogenicity	Multivariable coefficient (95%CI)	P- value
**Region**		
Amhara	4.26[1.46 to 7.12]	<0.0001
Central Ethiopia	0.78[-1.47 to 3.04]	0.29
Harari	1.86[-0.21to 3.93]	0.37
National [1]	2.88[0.70 to 5.06]	<0.0001
Oromia	1.71[-0.02 to 3.45]	0.49
Sidama	1.62[-0.57 to 3.81]	0.21
SWEPR	1.59[-0.63 to 3.79]	0.16
Tigray	1.84[-0.18 to 3.79]	0.43
Addis Ababa ®	1	
**Study period**		
[1]SDGs	-0.21[-1.58 to 1.16]	0.19
[2]MDGs®	1	
**Diagnosis criteria**		
[3]WHO®	-0.53[-2.08 to 1.03]	0.24
[4]SSA®	1	
**Study design**		
Case control	-0.58[-4.10 to 2.95]	0.18
Cohort	0.91[-0.75 to 2.58]	0.21
Cross sectional	1	

®*reference for the dependent variable*

[1] Study conducted in two and more regions; [2] sustainable development goal; [3] Millennium development goal **;** [4] World health organization ; [5] Sub Saharan Africa
